# Genetics in Ophthalmology

**DOI:** 10.1155/2018/4608946

**Published:** 2018-08-29

**Authors:** Lev Prasov, Stephen T. Armenti, Virginia Miraldi Utz, Julia E. Richards, Robert B. Hufnagel

**Affiliations:** ^1^Ophthalmic Genetics and Visual Function Branch, National Eye Institute, National Institutes of Health, 10 Center Drive, Bethesda, MD 20892, USA; ^2^Department of Ophthalmology and Visual Sciences, W. K. Kellogg Eye Center, University of Michigan, 1000 Wall St., Ann Arbor, MI 48105, USA; ^3^Department of Ophthalmology, Abrahamson Eye Institute, Cincinnati Children's Hospital Medical Center, 3333 Burnet Ave., No. 4008, Cincinnati, OH 45229, USA; ^4^Department of Epidemiology, University of Michigan, 1415 Washington Heights, Ann Arbor, MI 48109, USA

The clinical and molecular diagnostic evaluation of patients with heritable ocular disorders has evolved immensely since the first retinal dystrophy genes were cloned and sequenced in 1988 [1]. In addition, the modalities for studying the pathogenesis and future therapeutics for genetic eye disorders are constantly advancing. Through thoughtful consideration and detailed examination, imaging, electrophysiology, and family history, ophthalmologists and geneticists can select cytogenetic testing that confirms the clinical diagnosis in more than 50% of patients with retinal dystophies [2]. However, our understanding of disorders with complex inheritance as well as some Mendelian eye conditions is still in need of further investigation. For these unsolved traits, whole-exome and whole-genome sequencing provides a route for further investigation to identify a causative gene. This special issue of the *Journal of Ophthalmology* highlights the range of clinical and genetic testing modalities both for common and rare disorders, as well as complex and Mendelian traits.

Classic familial studies are highlighted in several studies presented in this issue. Wang and colleagues present a 4-generation family with a novel loss-of-function frameshift mutation in *PAX6*. The seven affected family members highlight significant intrafamilial ocular phenotypic variability. The authors propose mechanisms for phenotypic variability including interaction with and expression of other transcriptional factors involved in embryonic development, as well as variations in transcription and other epigenetic factors involved in *PAX6* expression. Falfoul et al. described two branches of a consanguineous Tunisian family harboring two *ABCA4* alleles, where different allelic combinations all led to cone-rod degeneration. They describe a novel complex structural *ABCA4* variant that has not been previously reported. In the paper by Smaragda et al., nearly 60 Greek patients with Stargardt disease were genotyped for *ABCA4* single-nucleotide and copy-number variant alleles, and almost 90% were detected to have pathogenic alleles. Surprisingly, the two most prevalent alleles were alleles with mild phenotypic expression. Beyond characterizing variant-level prevalence in specific populations, these studies may provide insight into predicting disease burden and progression in individuals based on their geographical origin.

Genetic and phenotypic heterogeneity are two important ideas that are continually discussed in this volume. A review paper by Carricondo et al. presents the clinical features of nanophthalmos and discusses the genetic factors implicated in this disease. Both autosomal dominant inheritance and autosomal recessive inheritance of nanophthalmos have been reported, and a fair amount of genetic heterogeneity is suggested by the finding so far of five nanophthalmos genes and two nanophthalmos loci. Phenotypic heterogeneity is reflected in the fact that four of the five known nanophthalmos genes (MFRP, PRSS56, CRB1, and BEST1/VMD1) can cause phenotypes other than nanophthalmos, although that level of heterogeneity has not yet been reported for nanophthalmos gene TMEM98. Sisk and colleagues present three patients who share a common phenotype of peripheral cone dystrophy (PCD), a condition in which the fovea and central visual acuity are preserved, but parafoveal photoreceptors undergo atrophy-accompanied macular vascular attenuation. The authors have redefined the clinical features to also include a nonprogressive course, normal choroidal thickness in areas of atrophy, and large affected regions giving a “bifocal” atrophic experience in 2 of 3 patients. The authors propose that genotypic heterogeneity and/or environmental influences may contribute to a common phenotype, as whole-exome sequencing failed to identify a common gene.

Association and gene expression studies have significantly advanced our understanding of complex traits. In the paper by Mingzen et al., the association of genes in the high-density lipopoprotein metabolic pathway with polypoidal choroidal vasculopathy (PCV) is explored. The authors found that 7 polymorphisms in genes in this pathway increase susceptibility to PCV, suggesting that the misregulation of this lipoprotein metabolism may be involved in the pathogenesis of this condition. Banevicius and colleagues evaluated the risk for optic neuritis in patients suspected to have impaired arachadonic acid (AA) metabolism and increased Th17 cell regulation. The authors show that single-nucleotide polymorphisms (SNPs) in CYPF2, a cytochrome P450 enzyme suspected to impair AA metabolism, were more frequent in men with optic neuritis (ON) and multiple sclerosis (MS) and that serum inflammatory cytokines (IL-17a) are elevated in patients with ON and MS. These results suggest that these factors may be associated with predisposition to ON and MS. In the paper by Stafiej et al., the authors explore the levels of TGF-beta2 and VEGF-a expression in epiretinal membranes (ERM) and internal limiting membranes (ILM) from vitrectomy specimens. They identify that TGF-beta2 and VEGF-a, factors associated with angiogenesis and wound healing, are upregulated in ERM as compared to ILM in all vitrectomy groups. Lumi and colleagues evaluate the frequency of SNPs in patients with and without proliferative vitreoretinopathy (PVR) to further validate whether polymorphisms in specific growth factors or cytokines may help stratify patients at risk for subsequent PVR after vitrectomy. Here, the authors suggest that although the variability in the distribution of SNPs across European subpopulations is significant, there are specific SNPs that occur with higher frequency in patients with PVR after vitrectomy compared to healthy controls in both interleukins and tumor necrosis factor genes. Drs. Lahola-Chomiak and Walter review the molecular genetics of pigment dispersion syndrome and pigmentary glaucoma, highlighting the clear genetic components of these disorders but the absence of clear single-gene mutations. Animal studies have demonstrated a role of melanosome function in the disease mechanism, but the precise pathogenic role of these genes in humans remains to be determined.

Epigenetic mechanisms also play a substantial role in gene regulation and the pathogenesis of ocular disease. In the paper by Chansangpetch et al., the authors explore the DNA methylation status of trabeculectomy specimens from primary open-angle glaucoma, primary angle-closure glaucoma, and secondary glaucoma patients, as compared to control specimens. They identified differences in the methylation status of Alu elements among glaucoma specimens as compared to controls, suggesting that methylation patterns may be associated with glaucoma pathogenesis.

The mixture of clinical ophthalmology and ophthalmic genetics found in these papers reflects the dawning role of precision medicine in ophthalmology. Diagnosis and medical management in a successful ophthalmic genetics practice requires expertise found in ophthalmology, medical genetics, genetic counseling, clinical molecular genetics, and often pediatrics. The team must apply rapidly changing molecular advances in the recent literature to patient care. Because so few individuals are trained and board-certified in all or most of these specialties, a multidisciplinary clinic is a viable alternative to have a single destination for families with inherited ocular disease. Another benefit to the combined clinic model is added power to interpret next-generation sequencing results and added education between specialties. As clinical molecular genetic testing options expand, we expect to see an increase in patients with disorders that cannot be diagnosed by gene panels or chromosomal technologies alone. As the range of molecular diagnoses delineate the list of clinical diagnoses into gene-specific diseases, involving medical and molecular geneticists for interpretation and second opinion of variants of uncertain or unknown significance may be crucial. It will help families and providers guide reproductive planning, medical and surgical interventions, and testing and surveillance of other family members where appropriate.

Optimally, the scope of testing will include known disease genes related to phenotypes. However, as the cost of whole-exome and whole-genome sequencing becomes more affordable, these technologies will be more frequently adopted. While analyzing with the intention to determine the primary cause of a condition, these methods will also yield additional information about variants of known or unknown significance (VOUS) without relation to the phenotype or desired diagnosis. So far, the American College of Medical Genetics has released a recommendation to counsel about unrelated results found in 59 genes that are medically actionable [3]. Perhaps more importantly, seeing the patient in a multidisciplinary clinic prior to ordering exome sequencing will aid in fully comprehending the family's intention and desire either to learn these unrelated risks or to blind themselves from knowledge of conditions for which no treatment exists.

In summary, this issue highlights the importance of genetics in every clinical specialty in ophthalmology. In this era of rapid genomics advancements, we face the challenge of interpreting and explaining complex testing options and results to patients. Thus, a collaborative, multidisciplinary approach is needed to provide comprehensive and informed care to patients. With recent advances in testing modalities, it will become increasingly important and complicated to obtain, distill, and present results of large datasets to our patients. Genetic testing has become more prevalent for identifying disease risk and for prognosis and is increasingly becoming used for understanding disease mechanism and developing new treatments. This in turn is creating important new opportunities that will call for every specialty in ophthalmology to join in this multidisciplinary clinical/genomic perspective on diagnostic and treatment choices. As our knowledge and experience expands, the role of genetics in ophthalmology will continue to grow in the years to come.

*Lev Prasov* *Stephen T. Armenti* *Virginia Miraldi Utz* *Julia E. Richards* *Robert B. Hufnagel*
